# Fundamental physics, existential risks and human futures

**DOI:** 10.1098/rsta.2023.0376

**Published:** 2025-05-08

**Authors:** Adrian Kent

**Affiliations:** ^1^ DAMTP, University of Cambridge, Cambridge, UK

**Keywords:** quantum foundations, gravity, consciousness, existential risk, human futures

## Abstract

Over the past 25 years, I have been involved in some intriguing developments in the foundations of physics, exploring the quantum reality problem, the relationship between quantum theory and gravity and the interplay between consciousness and physical laws. These investigations make it plausible that we will find physics beyond quantum theory, potentially including both new evolution laws and new types of measurement. There is also a significant chance they could have a potentially transformative impact on information processing and on the development of and our future with AI.

This article is part of the theme issue ‘Science into the next millennium: 25 years on’.

## 1. 25 years on

In my 2000 contribution [1], I focused on two central themes: the unresolved quantum measurement problem and the elusive scientific theory of consciousness. I argued that the quantum measurement problem is a genuine and fundamental problem in theoretical physics, and against the idea that the once-orthodox Copenhagen view of quantum theory gives a consistent and satisfactory metaphysics. We need either a reformulation of quantum theory or a deeper theory that gives a unified account of microscopic quantum physics and macroscopic (quasi-)classical physics. I pointed towards the dynamical collapse models developed by Ghirardi *et al*. [2,3] as the then most promising ideas in this direction. I also argued that we are lacking a fundamental scientific theory of consciousness that fits with the rest of theoretical physics as we understand it. The arguments I summarized go back to William James [4]; relevant reasons to be sceptical of reductive materialism go back at least to Democritus (see [5]); David Chalmers [6] gave arguments for a hard problem of consciousness, with which this stance also aligns.

In summary, we cannot claim a complete account of physics without including consciousness, since it gives all the evidence for our physical theories. We can nonetheless get an (apparently, presently) completely satisfactory account of material physics by invoking the principle of psycho-physical parallelism, according to which every conscious perception corresponds to physical events or processes in (presumably, generally) our brains, which is broadly supported by a wealth of neuroscientific data. But psycho-physical parallelism combined with standard material physics implies consciousness is an epiphenomenon. This makes it hard to understand why it is there at all, in a universe that would be equally logically consistent without it, and particularly hard to understand how it has all the properties that make it appear an evolutionarily finely honed survival mechanism, since—if genuinely epiphenomenal—it has no independent effect on the material world.

Both arguments invited readers to consider problems with what I took to be the mainstream views—some version of Copenhagen quantum theory and some materialist view of consciousness that denies any fundamental hard problem—and to take alternatives seriously. This only mildly assertive approach reflected genuine adogmatism:^1^ not everything we see as a fundamental scientific problem is necessarily solvable; every perspective on consciousness, in particular, is so problematic as to seem incredible. Still, my strong hunch was (and still is) that experiment will one day reveal some deeper physics underlying quantum theory. I also think a truly unified and complete description of nature will include consciousness as a fundamental natural phenomenon somehow—in ways we probably have not begun to conceptualize—related to mass, electromagnetism, gravity and the rest.

We are invited in this volume (I take it) to discuss how far our visions have been realized, and if, how and why they have changed. Space precludes reviewing much interesting and relevant work by others, so this will necessarily be very much a personal perspective.

## 2. What actually is the mainstream view of quantum theory?

What do physicists really think about quantum theory? Polls taken at quantum foundations conferences offer some clues, though they come with their own biases. The data in [7] suggest that the Copenhagen interpretation was still highly influential in 2013, as was the many-worlds interpretation. My impression is that in some significant physics research communities (for example string theory and cosmology) many-worlds ideas now dominate. It should be stressed though that ‘the Copenhagen interpretation’ and ‘the many-worlds interpretation’ have increasingly become umbrella terms, characterizing broad stances about fundamental physics rather than precisely formulated theories. In particular, there are many, many incompatible ideas (e.g. [8,9]) about what many-worlds quantum theory might mean. From time to time I have pointed out problems with several of the most prominent, including co-editing a volume [9] where I succeeded, at least, in persuading colleagues to frame the title as a question.

Without rehearsing all the arguments pro and con, let me touch on just one issue raised in [8] here. Whatever their other problems, standard ‘one-world’ versions of quantum theory make clear, scientifically testable statements about sequences of the apparently random outcomes of quantum experiments. We can understand these in terms of Kolmogorov’s notion of algorithmic compressibility. In this language, the hypothesis that a two-outcome experiment has a 1/3 probability of giving outcome 1 means that if you repeat the experiment many times then (i) you expect to be able to compress the list of N outcomes to a list of length NH, where H=−1/3log⁡(1/3)−2/3log⁡(2/3)<1 is the Shannon entropy of the outcome probability distribution, (ii) you do not expect to be able to compress it significantly further. If either prediction fails, then you should lose credence in the hypothesis. Many-worlds quantum theory gives a different hypothesis: all possible sequences of outcomes will really occur and be observed by a future successor of yours in some world. All these successors are equally real: none of them has a stronger claim to be ‘you’. If ‘you’ repeat the experiment many times, you are generating many successors observing different sequences, each of whom is unaware of the others. Whatever sequence any of them observes is consistent with the theory’s prediction about reality, which is thus unfalsifiable.

As my earlier article noted, the Copenhagen interpretation precludes a unified scientific theory encompassing microscopic and macroscopic physics, let alone cosmology. Many-worlds quantum theory proposes a unification—unitary quantum mechanics describes both microscopic and macroscopic, and so macroscopic superpositions persist. But its basic premise precludes confirmable or refutable scientific theories, and this seems impossible to avoid given the basic hypothesis.^2^

The universe is a strange place; perhaps no unified theory can fully describe it. If there is one, it might not be scientifically confirmable by observers within it. But we have made great progress in understanding it by seeking theories that are progressively more unified *and* scientifically confirmable. Methodologically, it makes sense to continue with standard scientific assumptions that have proven so fruitful, unless and until we have compelling reasons to think we have reached the limits of their validity. My guess is also that this will succeed: I would find a universe that completely resists scientific investigation, or one that can only be described by a patchwork of partial and incompatible theories, less surprising than one that holds out so much hope for science and unification, yet ultimately dashes these hopes.

## 3. Three paths to physics beyond quantum theory

### (a) Beables and new dynamics

#### (i) Beables

As already noted, the Copenhagen interpretation gives no precise way of unifying microscopic and macroscopic physics, which we need. Its implications were articulated most precisely by Bell [10,11]. We need a mathematical formalism characterizing what, exactly, quantum probabilities are probabilities of. As Bell put it, quantum theory is presented as a theory of observables, without telling us who or what qualifies as an observer. We need, he argued, a theory of *be*-ables [10], mathematically well-defined entities that characterize physical reality, within which observers, macroscopic objects, galaxies and so on are defined. On this view, none of the latter plays a fundamental role or necessarily has a precise definition, but the beables do and must. Mathematically, they define the sample space for quantum probabilities.

Bell pointed to de Broglie–Bohm theory [12,13] and dynamical collapse models [2] as interesting examples of beable extensions of quantum theory. Much theoretical effort has gone into developing dynamical collapse models in the last 25 years, but there remains no satisfactory relativistic field-theoretic version. Even more effort has gone into devising and implementing experiments to test the most studied models (Ghirardi–Rimini–Weber–Pearle (GRWP) mass-dependent spontaneous localization [3]), narrowing the parameter window [14] in which they remain viable to the point where (even if we set aside most people’s initial low credence) it would begin to seem a little conspiratorial if nature had chosen model parameters in the relatively small ranges that technology does not yet allow us to test. So I tend now to see GRWP models to date as interesting and well-motivated modifications of quantum theory that are not only very likely incorrect, but probably will not even turn out to be going in the right direction, yet should encourage us to explore further. De Broglie–Bohm theory, similarly, has no satisfactory relativistic field-theoretic version and, in its usual formulation, is not experimentally distinguishable from Copenhagen quantum theory.^3^ It also feels, in comparison to GRWP models, clunkily mathematically hybrid, combining the mathematical formalism of quantum theory with classical trajectories of point-like particles in a way that lacks the elegance we have come to expect from successful new physical theories.

So we need better beable theories. One idea I have been developing [16,17] combines insights from several approaches to quantum theory, including collapse models, the physics of decoherence and ideas in which initial and final states both play a fundamental role. The essential idea is that, in (fairly standard) cosmological models in which the universe expands forever, once we have a theory of the initial conditions and the unitary dynamics, a complete beable description of reality could be relatively simply and elegantly reconstructed from the asymptotic late time state of the quantized electromagnetic or gravitational field. This offers the hope of an explicitly relativistic model that (unlike de Broglie–Bohm theory) fits naturally with the framework of relativistic quantum theory. Of course, even if such a model is fully developed, it may not be correct. But it illustrates at least there is significant scope for new beable theories.

#### (ii) New dynamics from beables

Even an elegant beable model built thus on standard unitary quantum theory has a counter-intuitively unaesthetic feature: the beables are the fundamental building blocks of reality, yet inert. The mathematics of quantum theory defines the space of beables *and* the probability distribution of beable configurations. But it need not. We can define consistent theories [18] in which the probability distribution of a beable configuration depends on intrinsic properties of that configuration as well as on the quantum dynamics, even if we continue to restrict ourselves to standard quantum theory, in which those dynamics are determined by the initial state and a Hamiltonian that determines its evolution. Most theories defined in this way will look rather ad hoc—but the universe only needs one (which might appear elegant only after reframing, or, more likely, which might be only an approximation to an elegant underlying theory). The problem, if this line of thought is fruitful, is finding it, starting as we do from standard quantum theory.

Happily, framing the problem points not only to a solution but also to a vital scientific project that is motivated whether or not one takes beables seriously. A ‘beable-guided’ theory [18] that modifies standard quantum theory tells us that the probabilities of some sequences of events are different from those we obtain from the initial conditions and the quantum evolution laws. This is not because we have the wrong initial conditions or evolution laws, but because (in such a theory) initial conditions and standard evolution laws do not suffice as a description of the universe. One way of picturing this [19,20] is that the apparently random dice determining quantum events are actually collectively (not individually) biased to steer the universe towards some evolution paths and away from others. We do not know where to look for evidence of these ‘hodological’ (from *hodos*, Greek for path) models at the level of fundamental beables, absent a clear intuition about the form of a fundamental ‘beable-guided’ theory. But we can, and should, look for evidence that large-scale phenomena—in particular, cosmological events—are better described by non-standard hodological modifications of quantum theory than by standard quantum theory.

A common initial concern is that this takes us beyond the realm of science. In principle, one could devise a beable-guided theory that steers the universe towards any evolutionary path; in particular, it could steer the universe towards the very specific path we have observed, including the distribution of stars and galaxies and even the specific evolutionary history of life on Earth. Suggesting that things turned out the way they have because one has a theory that says (post hoc) in precise detail that they must have certainly would not contribute to science!

As in contrasting one-world and many-worlds quantum theory, algorithmic compressibility, specifically its development by Solomonoff [21] into an algorithmic formulation of scientific induction, shows us how to separate scientifically empty models from fruitful ones and how to work towards confirming or refuting the latter. Formally, the key idea [20] here is to use the principle of minimum description length for hypothesis identification [22], according to which the best hypothesis to fit the data is the one that minimizes the sum of the length of the program required to frame the hypothesis and the length of the string required to characterize the data given the hypothesis. Informally, cosmological models that modify quantum evolution relatively elegantly, using simple additional rules with few parameters, deserve some credence, and can and should be tested against the standard paradigm. Models that spell out in fine detail how the universe evolved are given essentially no credence, because specifying them takes a very large amount of information (in the extreme case, recapitulating the actual history of the universe). There are only a finite number of models of any given description length, and we can confirm or refute these with a finite amount of observational data.

Recent hints [23] that dark energy may vary over time and space—a feature that could easily be incorporated into a hodological model, which might or might not require few parameters to fit the data well—add significantly to the motivation for this program. Granted, cosmological data are hard to interpret, and cosmologists continue to argue about the strength of evidence for or against standard cosmological theories. Also, Solomonoff induction is defined with respect to a given computing model and language, which leaves some (albeit finite) uncertainty about how to apply it in practice to science framed in a mixture of human language and mathematics. These issues make implementing this program somewhat more complex, but do not alter the key point: we can certainly produce simple hodological cosmological models that do not fit into the Newtonian initial-causes-suffice paradigm, and we should be trying to test that paradigm against them. Even if one thinks of these models only as foils rather than serious contenders, they allow us to parametrize and make precise the extent to which we have confirmed the standard paradigm.

**Figure 1.**
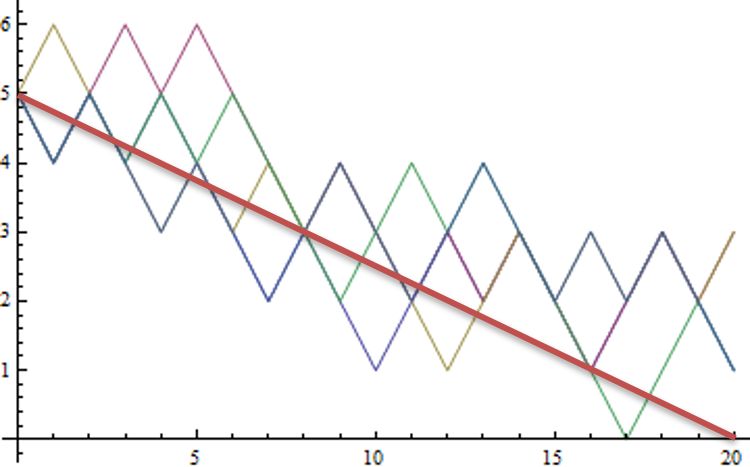
Effect of a hodological path-guided law in a simple Ehrenfest urn model (from [20]). Ten numbered balls are initially divided equally between two urns. At each of 20 steps, a random ball is chosen and moved to the opposite urn. The standard model has no other rule, and produces fluctuations around equidistribution. The rule illustrated here weights the probability of any sequence depending on its mean square separation from the line n=5−(t/4) (red line), producing typical sequences shown by the other coloured paths. The chance of any such path arising by chance in the original model is so small that, if it were observed, we would inductively infer a rule similar to the one given. This simple model illustrates how path-guided laws could steer the universe to favour some paths, challenging the independent random time-step evolution predicted by standard quantum theory.

An illustration of the effects of a simple hodological rule in a simple toy model is given in figure 1. As discussed there, if we saw such effects, our credence in a hodological rule similar to the one given would be greatly increased, and our credence in a standard Newtonian rule correspondingly diminished.

### (b) Gravity

The second reason for looking beyond quantum theory is that we still do not have a conceptually satisfactory quantum theory of gravity, nor any evidence that gravity is quantized. Many physicists have come to believe we do not need evidence because there is no consistent way of combining a classical theory like general relativity with quantum theory. The argument here is that any coupling between classical and quantum degrees of freedom would allow us (at least in principle) to obtain ‘too much information’ about quantum states, implying faster-than-light signalling. However, arguments for this [24] have been refuted [25]. In fact, it turns out there are very many logically consistent ways of extending quantum theory that allow non-quantum measurements [26–28] that give more information about quantum systems than standard quantum measurement theory permits.

More recently, it has been suggested [29] that such measurements are inconsistent with the other postulates of quantum theory. This argument relies not on faster-than-light signalling but on the related but distinct so-called quantum no-signalling principle, which says that operations on one isolated subsystem should have no measurable effect (at any time) on another. This is sometimes expressed as the principle that physical influences must have physical carriers. In the context of general relativity, though, there are no truly isolated subsystems: matter anywhere influences matter everywhere through its gravitational field, i.e. its action on space–time, which indeed serves as a physical carrier for physical influences. It is certainly possible to imagine that, in some theory unifying quantum theory and general relativity, additional (presently unspecified) degrees of freedom carry additional information alongside, and presumably intertwined with, the information that space–time carries in special relativity, and that non-quantum measurements use this additional information. For this reason, I place higher credence than most of my colleagues in the possibility that future physics will go beyond standard quantum measurement theory^4^—which would potentially have a dramatic effect on quantum computing and quantum cryptography as well as fundamental physics (see below).

These arguments have become much more pertinent because of one of the most exciting recent developments in fundamental physics, the realization that the quantum nature of gravity can be tested in the low-energy regime by finding evidence for or against gravitationally induced entanglement or other effects that are predicted by all standard quantum gravity theories but not by theories in which the gravitational field is classical. The key idea was first discussed by Bose *et al*. [30] and independently by Marletto & Vedral [31]. Although the relevant experiments are not feasible with present technology, they are much closer to feasibility than previous proposals relying on Planck energy effects. They have added impetus to work (e.g. [32,33]) on specific hybrid theories in which a classical gravitational field is coupled to quantum matter, as well as to the general possibilities just mentioned. Debate continues (e.g. [34–36]) over exactly what these experiments or variants [37] would test. The initial hope that they might give a clean and definitive test of whether gravity is quantum, with essentially no additional assumptions, seems over-optimistic. However, they would either refute or confirm the most interesting alternative types of model identified so far, and hence either refute or give strong new evidence for quantum gravity. Moreover, simpler versions of the experiments, which are likely to be feasible sooner, will already refute or confirm some interesting alternatives [38,39].

### (c) Consciousness

Writing about consciousness and physics 25 years ago felt risky, and it felt positively foolhardy to promote William James’ argument [4] against consciousness being an epiphenomenon, a by-product with no causal influence. As James pointed out, if so, it is implausibly fine-tuned, appearing very well developed to reinforce evolutionary advantageous behaviours while actually producing a mere narrative, laden with attractions and aversions that appear to motivate but have no independent effect on the material organism. It has been heartening to see an explosion of interest over the last couple of decades in consciousness as a fundamental physics problem, guided, inter alia, by David Chalmers’ beautifully lucid expositions [6] of the motivations for and difficulties with every line of thought, stimulated further by attempts at an ‘integrated information theory’ [40,41] of consciousness, including a revival of interest (e.g. [42,43]) in the physical arguments for panpsychism promoted by Russell [44], Eddington [45] and others, and also including a serious proposal to test the implications of James’ argument by looking [46] for deviations from quantum theory on a quantum computer.

Let me summarize where I think we currently stand. Some argue that creatures that have evolved to interpret and reason about the world and their own interactions with it necessarily must be conscious, and that we, with our brains, necessarily must be conscious in the way we are. They deny the logical possibility of philosophical zombies, inhabiting an alternative universe with a material world identical to ours (starting from the same state, following the same laws, with the same random quantum events) but without any consciousness. To many others, including me, this is incoherent: it is an objective fact that I (and I am sure you, and almost all animals) have subjective experiences, but not a fact that follows from quantum theory or general relativity. It is logically consistent, given what we understand about physics and consciousness at present, to imagine a universe with the same material evolution in which those experiences were different, or absent.

Dialogue between these camps can be difficult. Many people seem to join one early in their intellectual life and find their conceptual framework allows them to translate what the other camp seems to be saying as some form of obvious error or failure to appreciate elementary points. Only a minority come to appreciate that their own position does actually, like every stance on consciousness, have difficulties. For example, accepting James’ argument against epiphenomenal consciousness, I am still struggling [47–49] even to sketch the possible form of a non-epiphenomenal model that could satisfactorily explain why our consciousness appears evolutionarily well-adapted.

When thoughtful people disagree so radically on a fundamental point, on which every articulated position appears problematic on careful analysis, one ought rationally to broaden one’s credence distribution. James’ argument, arguments for panpsychism or panprotopsychism, and arguments for dualism all suggest there should be new dynamical laws associated with consciousness. Arguments for epiphenomenalism or illusionism suggest there should not. So, arguably, does the fact that current material physics does indeed describe the dynamics of living creatures very well: we have seen no dramatic anomalies, so any new dynamical effects would have to be very subtle. Cashing all this out leaves me with significant, if not overwhelmingly high, credence in new laws to be found.

## 4. Synthesis

Three distinct threads—quantum reality, gravity and consciousness—give completely different motivations for exploring physics beyond quantum theory. We also have a panoply of examples of ways in which quantum theory can consistently be modified. These threads could weave together and converge into a single tapestry, revealing a universe far richer and more interconnected than most physicists have so far imagined. For example, the unification of quantum theory and gravity could involve a hodological model whose predictions are most easily tested by cosmological observation. Or, as Penrose has suggested [50], resolving the quantum measurement problem might involve collapse mechanisms associated with both gravity and consciousness. Or even, much more speculatively, fleshing out arguments by Nagel [51], the emergence of consciousness in the universe might need a hodological explanation. But the arguments need not connect and of course they need not all be right (even if one is).

We can and should test separately the ideas motivated by each line of thought. Much effort is now going into developing the technology needed to test quantum gravity against alternatives, and into theoretical ideas that might allow easier or different tests. Proposals that would test in some way the relationship between consciousness and quantum theory include: the aforementioned searches for anomalous outputs from quantum computers; work on collapse models that implement Wigner’s hypothesis that consciousness collapses wave functions, using explicit mathematical models for measures of consciousness; experiments involving living organisms and Bell experiments with well-separated human observers directly observing the outcomes in each wing. I hope to build a collaboration, testing a good range of hodological cosmological models.

We should take on board the broader moral, though: new physics underlying quantum theory could manifest itself in many ways, most of which we likely have not yet envisaged. This motivates a much broader and more systematic ‘stress-testing’ of quantum theory in untested regimes, and an active search for dynamical anomalies. For me, this is the most important project in science. We could finally go beyond the Newtonian paradigm; we could find a deeper theory underlying quantum theory and relativity; we could gain new fundamental insight into the nature of consciousness and our relation to the universe. And we could also possibly transform information technology and the evolution of intelligence on (and beyond) Earth, as §4a discusses.

### (a) Fundamental physics and human futures

My earlier essay closed with the comment that the Editor of the corresponding volume in 2999 would very likely be able to solicit contributions from extraterrestrial and/or AI colleagues. Today, that future feels much closer. Like most, I did not foresee that AI contributions would already be possible (if arguably not yet quite as interesting as human ones) in 2025. It now looks very plausible that there will be general-purpose superhuman artificial intelligence in the coming decades. Not everyone is persuaded (e.g. [52]). On the other hand, many experts think it will be much sooner [53]; indeed, serious efforts seem under way to promote a Manhattan-style project to achieve it in the next few years. Let us accept that the hypothesis of artificial general-purpose intelligence in a few decades deserves significant credence: it does not ultimately matter so much for the argument whether the credence is 1% or 99%. I am now going to set out arguments, with the caveat that most need much further and more thoughtful analysis. This is a manifesto for a research programme, a key part of which is to scrutinize the plausibility of each hypothesis.

We start with the hypothesis that humans have perhaps 30 years (and maybe significantly fewer) left to steer fundamental physics research before AI takes over. The only possible discoveries that could have a longer term impact on our well-being are those that affect the development of AI itself. Extending the theory of elementary particles would be scientifically fascinating, of course, but seems very unlikely to have any technological impact in 30 years. Nuclear physics did, of course, have an enormous impact. A transformatively new way of generating energy could have some impact, for better or worse—for example the cold fusion dreamed of by mavericks might, hypothetically, allow effectively unlimited cheap clean energy (and so cheaper and less environmentally damaging AI data centres) but also cheap and potentially devastating new radiation weapons. But (pace [54]) there seems no plausible reason to expect such a development. The progress of sub-atomic physics since the 1930s and essentially all serious theoretical ideas about particle physics beyond the standard model suggest we should not expect new ways of liberating energy from any other development in this area either.

The other possibility that could profoundly impact the development of AI and other key technologies is a transformative development in information processing. This *is* a more plausible consequence of the possible new physics beyond quantum theory we have discussed. Quantum computing is believed to be more powerful than classical computing at some significant tasks, including factorization, simulation of quantum systems and (though with only a square root speed-up) searching. Many generalizations of quantum theory would, in principle, give models of computation that are significantly more powerful than quantum theory. For example, in models of computing using a perfect implementation of a nonlinear version of quantum theory we would have [55] P = NP, implying oracle-like computing power, with which obtaining the solution to what (in our current understanding is) an intractably hard problem would not be significantly harder than verifying a given solution [56]. It seems unlikely any post-quantum physics would give us the effectively infinite precision control that these models require, but not so implausible that it could give us a significantly stronger model of computing, and perhaps thus a significantly more powerful form of machine learning, than quantum theory does. It could similarly transform our understanding of more general physical learning machines [57].

We thus have a (perhaps small, though I suspect significantly larger than most physicists consider) probability p of a future fundamental physics discovery that would have a very large impact I on human welfare and on the future development of intelligence. To the extent that we think humans will be able to beneficially steer the development of AI (on which, to be clear, expert opinion ranges from hubris to despair) we should much prefer that this discovery is made by humans, in the phase where we are still designing AIs and integrating value alignment and guardrails, rather than by AI, in the phase where it is self-designing and any attempts at human steering have less if any effect.

One of the most significant developments of the last 25 years has been the academic mainstreaming of research and policy focused on this type of (maybe) low probability, (certainly) high impact existential risk and opportunity. In 2000, when I pointed out major flaws in risk assessments for the speculative hypothesis [58,59] that so-called ‘killer strangelets’ might be created in collider experiments and cause a catastrophic runaway reaction, I encountered much resistance from colleagues even to the elementary point [60] that one needs to evaluate risks in proportion to their impact as well as their probability, meaning that a probability bound of $2\times 10^{-5}$ on accidentally destroying the Earth gives a decidedly unreassuring bound of $10^{5}$ in expected present human lives lost. A truer measure [60] of the hypothetical catastrophe allows for future human lives lost as well as present, giving a bound (obviously with much more uncertainty) of something very roughly in the region of $\approx 10^{13}$ human lives.

Thanks to the work of the Cambridge Centre for the Study of Existential Risk, Oxford’s Future of Humanity Institute and partner organizations around the world, the naive arguments offered by CERN scientists [58] to the effect that ‘p is proven small, or at worst comparable to other natural extinction risks, so the experiment is safe enough’ would encounter much more formidable resistance today. This is not to say we would or should now be paralysed by each and every new risk hypothesis. The existential risk community have also popularized and addressed the problem of ‘Pascal’s mugging’, a term first coined by Elizier Yudowsky, in which a plethora of minuscule probability ultra-high-risk and/or -reward hypotheses threaten rational decision-making. Before being too swayed by purported bounds for expected lives or related quantities, we need, at least, to be confident in evaluating a lower bound for p, an upper bound for I and, crucially, a horizon scan of other hypotheses with similar or higher estimates of pI. In the case at hand, this means asking, inter alia: what should our credence in alternatives to quantum theory be? That, if there is a theory underlying quantum theory, it actually gives major advantages for computing and machine learning in principle? That it gives advantages that could realistically be exploited technologically in the next 30 years? That such a theory will be found in the next 50 years? That a more human-friendly form of future intelligence is significantly more likely if the discovery is made and exploited by humans? That careful answers to all of this leave the hypothesis high on the list of concerns worth addressing, given the several known significant existential risks (e.g. [14]) and many other hypothetical ones? While I lack definitive answers, the urgency and plausibility of these questions compel me to advocate for a larger scale, collaborative effort to explore them.

Provocatively, but seriously, I have set out in table 1 a list of the relevant hypotheses, my considered credences at present and their impacts if true. The credences may seem high to many readers. One reason for that is that I have quite high credence that the quantum measurement problem requires new physics, low credence that any version of many-worlds quantum theory can resolve it and also quite high credence in stances that suggest new physics associated with consciousness. If your credences in each of these are enormously different, it is worth considering that many thoughtful physicists differ, and asking how confident you should be that your reasoning and intuition are superior to theirs. (The reverse is true too, of course: this is why my credences do not approach certainty.) Another reason is the general point that historically, scientists have always tended to be overconfident in the current paradigm. Of course, even taking these points into account, credences can still reasonably differ substantially. Note though that even credences $10^{-3}$ smaller than mine would still produce very large values of pI and justify supporting research programmes, if Pascal’s mugging considerations turn out not to mandate downscaling the effective impact very substantially.^5^ The next decades may well determine whether humanity remains the primary driver of scientific discovery—or cedes that role to the very intelligences we create. The programmes I have outlined at least increase the chances that we retain more control, for longer, and propel science along a path better aligned with human values.

**Table 1.** Hypotheses, credences, impacts.

hypothesis	credence	impact
path-guided evolution laws	0.25	revolution in understanding quantum theory and cosmology
non-quantum gravity	0.25	revolution in understanding fundamental physics
‘post-quantum’ measurements (PQMs)	0.1	revolution in understanding quantum theory
PQMs give much more powerful computing:		
in theory	0.04	potential technological revolution
in medium term	0.01	technological and AI revolution
new evolution laws connected with consciousness (EC)	0.2	revolution in understanding mind–matter relation
EC allows an effective consciousness meter	0.15	policy and ethics revolution
EC gives much more powerful computing:		
in theory	0.12	potential technological and AI revolution
in medium term	0.01	technological and AI revolution

## Data Availability

This article has no additional data.

## Footnotes

^1^
Though also, I did not have tenure.

^2^
Though for some attempts, see [9].

^3^
Though it can be made so [15].

^4^
Renato Renner and I have bet on whether this will happen by 2040, staking 100 cases of wine (Renato) against one (me).

^5^
For comparison, Toby Ord [61] gave estimates of 10^−2^ for the risk of existential catastrophe arising from nuclear war or catastrophic climate change over the next century. These smallish estimates (now somewhat upgraded and downgraded, respectively [62]) certainly were not meant as arguments against devoting large resources to mitigating these risks.
